# A Thermopile Infrared Sensor Array Pixel Monolithically Integrated with an NMOS Switch

**DOI:** 10.3390/mi13020258

**Published:** 2022-02-04

**Authors:** Hongbo Li, Chenchen Zhang, Gaobo Xu, Xuefeng Ding, Yue Ni, Guidong Chen, Dapeng Chen, Na Zhou, Haiyang Mao

**Affiliations:** 1 Institute of Microelectronics of Chinese Academy of Sciences, Beijing 100029, China; lihongbo@ime.ac.cn (H.L.); zhangchenchen@ime.ac.cn (C.Z.); xugaobo@ime.ac.cn (G.X.); chenguidong@ime.ac.cn (G.C.); dpchen@ime.ac.cn (D.C.); 2 University of Chinese Academy of Sciences (UCAS), Beijing 100049, China; 3 Jiangsu Hinovaic Technologies Co., Ltd., Wuxi 214135, China; dingxuefeng@hinovaic.com (X.D.); niyue@hinovaic.com (Y.N.)

**Keywords:** thermopile infrared sensor array (TISA) pixel, dual-layer p+/n- poly-Si, monolithic integration, interleaved MEMS and CMOS processing, NMOS switch

## Abstract

In this article, we present the design, fabrication, and characterization of a thermopile infrared sensor array (TISA) pixel. This TISA pixel is composed of a dual-layer p+/n- poly-Si thermopile with a closed membrane and an n-channel metal oxide semiconductor (NMOS) switch. To address the challenges in fabrication through the 3D integration method, the anode of the thermopile is connected to the drain of the NMOS, both of which are fabricated on the same bulk wafer using a CMOS compatible monolithic integration process. During a single process sequence, deposition, etching, lithography, and ion implantation steps are appropriately combined to fabricate the thermopile and the NMOS simultaneously. At the same time as ensuring high thermoelectric characteristics of the dual-layer p+/n- poly-Si thermopile, the basic switching functions of NMOS are achieved. Compared with a separate thermopile, the experimental results show that the thermopile integrated with the NMOS maintains a quick response, high sensitivity and high reliability. In addition, the NMOS employed as a switch can effectively and quickly control the readout of the thermopile sensing signal through the voltage, both on and off, at the gate of NMOS. Thus, such a TISA pixel fabricated by the monolithic CMOS-compatible integration approach is low-cost and high-performance, and can be applied in arrays for high-volume production.

## 1. Introduction

The three-dimensional (3D) integration of micro-electro-mechanical system (MEMS) devices or microsensors with CMOS signal processing circuits on separate chips by stacked bonding and electrically connecting neighboring chips with through-silicon-vias (TSVs) offers significant advantages, such as a low cost, high performance, and multiple functions within a single package [1,2,3].

However, a large volume of high-power-density thermal accumulation, complex 3D architecture, diverse design tools and a high alignment accuracy all present technical challenges for 3D integration [4,5,6,7,8]. Although these challenges could be overcome by elaborate optimization, they inevitably lead to additional cost and technical risk. Thus, although various 3D integration schemes have been developed, only the heterogeneous integration of devices with different processes shows performance and cost advantages [9,10,11,12].

Compared to 3D integration, monolithic integration, which integrates MEMS sensors and CMOS ICs on the same substrate using consecutive or interlaced processing schemes, has shown great potential for MEMS devices fabricated by a monolithic integration process. By fabricating MEMS and CMOS on the same chip with a single process sequence, a system on chip with a high performance, small footprint, cost-effective packaging, little signal noise, and low cost can be obtained [13,14,15].

Monolithic integration of MEMS sensors and CMOS ICs can be categorized into three basic approaches: MEMS-first processing, MEMS-last processing, and interleaved MEMS and CMOS processing. In MEMS-first processing, all required processing steps for MEMS devices are achieved prior to CMOS processing. This method allows for very high-temperature processing of the MEMS materials, which is successfully applied in the high-volume fabrication of MEMS resonators and pressure sensors [16,17,18]. In contrast to the MEMS-first processing, in the MEMS-last processing, CMOS ICs are prepared before MEMS devices. MEMS-last processing makes it so that the CMOS design does not need to be modified and has greater design flexibility and shorter design cycles, for wide use in the commercial manufacture of displays, gyroscopes, accelerometers, and so on [19,20,21,22]. Interleaved MEMS and CMOS processing is achieved through a combination of MEMS processing steps performed before, after, or during CMOS fabrication. Interleaved MEMS and CMOS processing is suitable for MEMS devices by CMOS processes and offers the possibility of integrating high-performance MEMS devices together with CMOS ICs on the same substrate. This processing approach has been applied in various fields, ranging from pressure sensors and infrared sensors to neural probes [23,24,25]. Compared with the other two monolithic integration approaches, interleaved MEMS and CMOS processing can effectively reduce the number of masks to less than the sum of the numbers required for individual MEMS and CMOS ICs, respectively, and shorten the overall fabrication cycle.

As a side note, MEMS thermopiles sensors are used as important uncooled bolometers and pyroelectric detectors [26,27] and this type of sensor with high performance can be mass-produced by using standard CMOS processes [28,29,30,31]. This makes it possible for MEMS thermopiles and CMOS ICs to be integrated on the same chip by adopting interleaved MEMS and CMOS processing. Meanwhile, as one of the most basic elements in CMOS ICs, an NMOS has a switching function in its triode region and an amplifying function in its saturated region.

In this study, a dual-layer p+/n- poly-Si thermopile and an NMOS are integrated on the same chip using interleaved MEMS and CMOS processing. Herein, the NMOS is employed as a switch, the drain of which is connected to the anode of the thermopile, thus constructing a pixel in a thermopile infrared sensor array (TISA). In addition, compared to a separate thermopile, a thermopile integrated with an NMOS switch maintains a fast response, large sensitivity and high reliability. Therefore, such an approach is expected to be applied in the monolithic integration of MEMS TISAs and CMOS ICs.

## 2. Design Principle of the TISA Pixel

**Figure 1:**
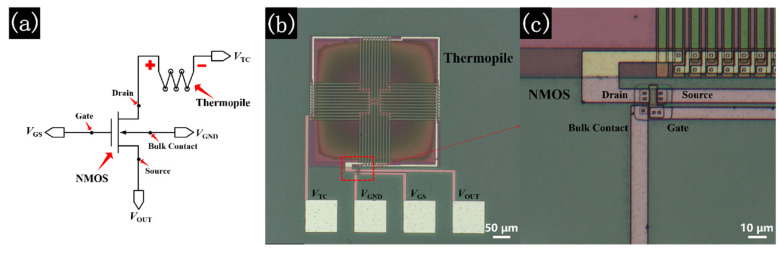
(**a**) Equivalent model of TISA pixel, (**b**) optical image of TISA pixel, (**c**) an enlarged image of NMOS.

Figure 1 demonstrates the equivalent model and optical images of a TISA pixel. In the pixel, the anode of the thermopile is connected to the drain of the NMOS. Based on the characteristics of NMOS, when *V*_GS_ > *V*_TH_, where *V*_GS_ is the gate voltage of NMOS and *V*_TH_ is the threshold voltage of NMOS, an electron layer is formed near the gate oxide layer in the substrate. Then, when a positive voltage (*V*_DS_) is applied between the drain and the source of the NMOS, there will be a drain-source current (*I*_DS_) going through the channel formed by the electron layer. Thus, such an NMOS can function as a switch that reads the drain voltage by controlling *V*_GS_.

By virtue of the transconductance of NMOS, when *V*_DS_ < *V*_GS_ − *V*_TH_, the NMOS operates in the triode region. Therefore, *I*_DS_ can be expressed as:

(1) $$I_{\mathrm{DS}}=\mu_{n}\cdot C_{\mathrm{OX}}\cdot \frac{W}{L}\cdot \left(V_{\mathrm{GS}}-V_{\mathrm{TH}}\right)\cdot V_{\mathrm{DS}}$$

Here, *μ*_n_ is the mobility of charge carriers, *C*_OX_ is the capacitance per unit area of the gate oxide, *W* is the effective channel width, and *L* is the effective channel length. When there is a thermopile sensing voltage (*V*_T_) and a bias voltage of the thermopile cathode (*V*_TC_) at the drain of NMOS and output resistance (*R*_OUT_), *V*_DS_ can be obtained:

(2) $$V_{\mathrm{DS}}=V_{T}+V_{\mathrm{TC}}-I_{\mathrm{DS}}\left(R_{T}+R_{OUT}\right)\;$$

For simplicity, the following equation was utilized:

(3) $$\mu_{n}\cdot C_{\mathrm{OX}}\cdot \frac{W}{L}\cdot \left(V_{\mathrm{GS}}-V_{\mathrm{TH}}\right)=a$$

By substituting Equations (2) and (3), respectively, into Equation (1), there is:

(4) $$I_{\mathrm{DS}}=\frac{a\cdot \left(V_{T}+V_{\mathrm{TC}}\right)}{1+a\cdot \left(R_{T}+R_{\mathrm{OUT}}\right)}$$

Output voltage (*V*_OUT_) can also be derived from the schematic:

(5) $$V_{\mathrm{OUT}}=I_{\mathrm{DS}}\cdot R_{\mathrm{OUT}}$$

The completed expression of *V*_OUT_ can be obtained by substituting Equation (4) into Equation (5):

(6) $$V_{\mathrm{OUT}}=\frac{V_{T}+V_{\mathrm{TC}}}{\frac{1}{a\cdot R_{\mathrm{OUT}}}+\frac{R_{T}+R_{\mathrm{OUT}}}{R_{\mathrm{OUT}}}}$$

From Equation (6), it is observed that as a is a product of mobility of charge carriers, capacitance per unit area of the gate oxide and channel width–length ratio, and *R*_OUT_ matches the high impedance input of the reading circuit, both of them are large values. Therefore, $\frac{1}{a\cdot R_{\mathrm{OUT}}}$ can be approximately ignored. Finally, the relation between the pixel output voltage and thermopile sensing voltage is obtained:`

(7) $$V_{\mathrm{OUT}}\approx \frac{R_{\mathrm{OUT}}}{R_{T}+R_{\mathrm{OUT}}}\cdot (V_{T}+V_{\mathrm{TC}})$$

Herein, the larger *R*_OUT_ is, the closer *V*_OUT_ is to real thermopile sensing voltage.

**Figure 2:**
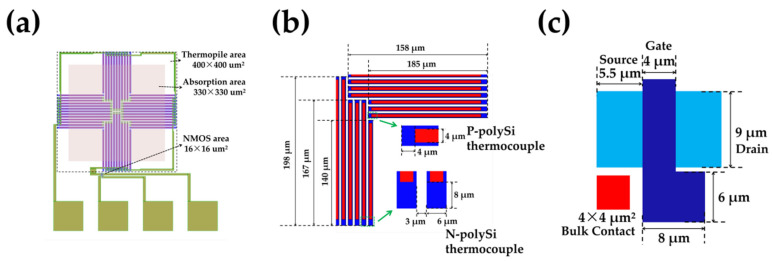
Schematics of (**a**) the TISA pixel, (**b**) the thermocouple and (**c**) the NMOS.

Figure 2 shows the schematics of the TISA pixel, the thermocouple, and the NMOS, respectively, that we designed. In the pixel, in order to maximize the number of thermocouples and place the hot junctions as close to the center of the absorption area as possible, the thermopile is designed with centrosymmetric structures and has an area of 400 μm × 400 μm and consists of 48 pairs of p+/n- poly-Si thermocouples with a 330 μm × 330 μm Si_3_N_4_ absorption layer. The NMOS has an area of 16 μm × 16 μm, whose effective gate width and effective gate length are, respectively, 4 μm and 9 μm.

## 3. Micro-Fabrication of the TISA Pixel

**Figure 3:**
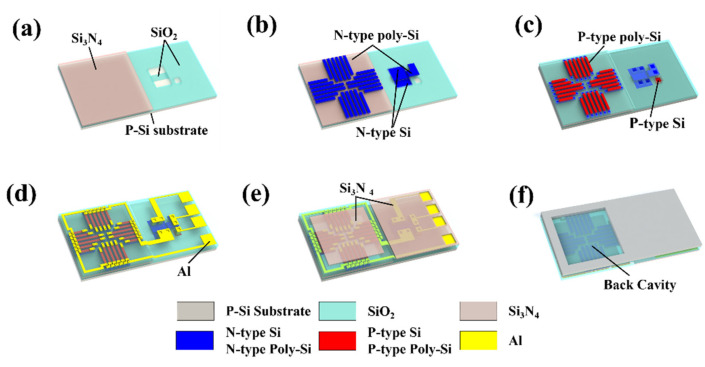
Fabrication processes of TISA pixel monolithically integrated with NMOS. (**a**) Deposition and patterning of silicon oxide and silicon nitride, (**b**) Poly-Si deposition, patterning and phosphorus ions implantation, (**c**) Poly-Si deposition, patterning and boron ions implantation, (**d**) Silicon oxide deposition and Al interconnection, (**e**) Deposition and patterning of silicon oxide, (**f**) Deep silicon releasing at the back-side.

Based on the aforementioned design of the TISA pixel, the device is fabricated based on an interleaved MEMS and CMOS processing. Figure 3 shows the fabrication process and Table 1 shows the roles of each process and each mask. First, a p-type ((100), 8–12 Ω·cm) silicon wafer is used as the substrate. Then, a silicon oxide layer is grown by wet oxidation and patterned, which is further used as the doping barrier for the NMOS.

With a silicon nitride layer deposited in the following step, the silicon oxide layer further functions as a supporting layer for thermocouples. Afterwards, a thin oxide layer grown by dry oxidation is employed as the dielectric layer at the gate stack of the NMOS, where the photopatterned parts are employed as the ion implantation windows of the source and the drain.

Taking this as a basis, the first polysilicon layer is deposited by using low pressure chemical vapor deposition (LPCVD), and then patterned and implanted by phosphorus ions to form the gate of the NMOS and the n-type thermocouple strips of the thermopile. At the same time, the drain and the source of the NMOS are also formed with the same ion implantation step. After that, a silicon oxide layer is deposited as an insulator between the stacked n-type and p-type thermocouple strips, which is followed by deposition, pattern and B^+^ implantation of the second polysilicon layer to form the p-type thermocouple strips and the bulk contact of the NMOS. Subsequently, rapid thermal annealing is performed to activate the injected ions. Later, a silicon oxide layer is deposited and etched to be employed as an insulator for the following Al interconnections. Then, another silicon oxide is deposited and patterned to form a thermal conductance layer between the absorption layer and the thermocouples. In this device, a silicon nitride layer is used as the infrared absorption layer. Finally, the thermopile structure is released by deep reactive ion etching (DRIE) from the back side, forming thermal insulation of hot junctions.

Based on the above processes, a TISA pixel integrated with an NMOS switch is obtained. In the fabrication process, several steps are simultaneously performed to respectively form the thermopile and the NMOS, such as ion implantation, and metal layer sputtering, as well as silicon oxide, silicon nitride, and polysilicon layer deposition. The fabrication process of thermopile and NMOS requires 9 and 7 lithography, respectively, while the monolithic integration process requires only 10. This method can be used for MEMS–CMOS integration to effectively reduce the number of process steps and the cost of subsequent assembly and packaging.

## 4. Results and Discussions

The fabricated structure was characterized by using SEM (Zeiss, AURIGA Compact, Oberkochen, Germany). Wafer-level testing of current–voltage (I-V) characteristics was performed using a semiconductor parameter analyzer (Keithley 4155B, Solon, OH, USA).

**Figure 4:**
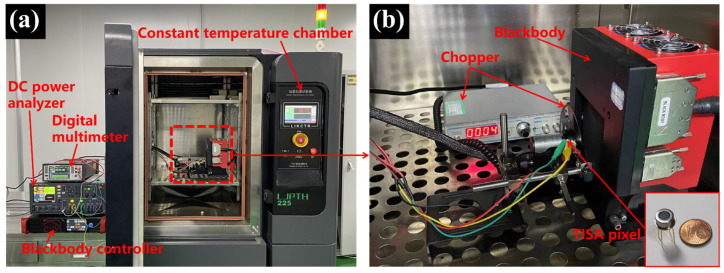
(**a**) The test system for a separate thermopile and a TISA pixel, (**b**) An Enlarged photograph at the blackbody position.

Subsequently, the devices were packaged in TO39 cans with 5.5–14 μm band-pass filters, and the cans were filled with nitrogen for reducing ambient noise, decreasing thermal conductance and enhancing light-wave transmittance. The test system for packaged devices is shown in Figure 4. A DC power analyzer (Keithley 6705C, Solon, OH, USA) provided two DC voltage sources. The current-–voltage (I-V), thermoelectric sensitivity and switching characteristics were measured using a semiconductor parameter analyzer (Keithley 4155B, Solon, OH, USA), a high-precision digital multimeter (Keithley DMM6500, Solon, OH, USA), and an IR blackbody (HGH, DCN1000H4LT, Igny, France). Meanwhile, in order to evaluate the performance of the TISA pixel integrated with the NMOS, we tested and compared it with a separate thermopile and a separate NMOS fabricated on the same wafer.

### 4.1. Morphology of the TISA Pixel

**Figure 5:**
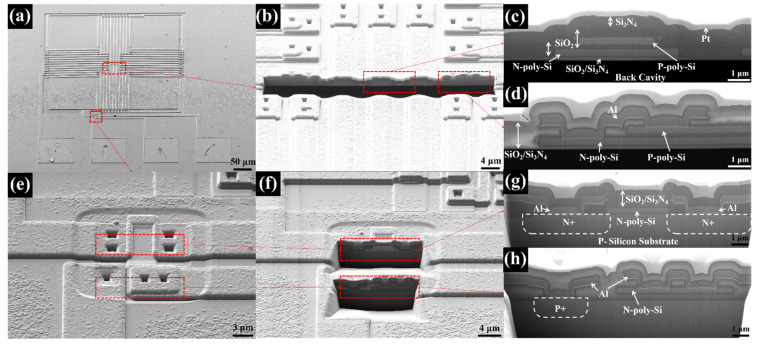
SEM images of (**a**) A TISA pixel, (**b**) A sectional position of a thermopile, (**c**) An enlarged image of a thermocouple, (**d**) An enlarged image of the hot junction, (**e**) An enlarged image of the NMOS, (**f**) Sectional positions of the NMOS, (**g**) An enlarged image of drain-gate-source structure, (**h**) An enlarged image of bulk contact and gate contact.

Figure 5 shows the SEMs of the TISA and the NMOS, the sectional positions of the thermopile zone and NMOS zone, and the local enlarged images at the sections. P-type and n-type polysilicon employed as the thermocouples and the gate of NMOS can be e observed. Each layer is stacked in turn, and the steps between layers are well covered. Al completes the connections and extractions of the functional layers.

### 4.2. Characteristic Analysis

**Figure 6:**
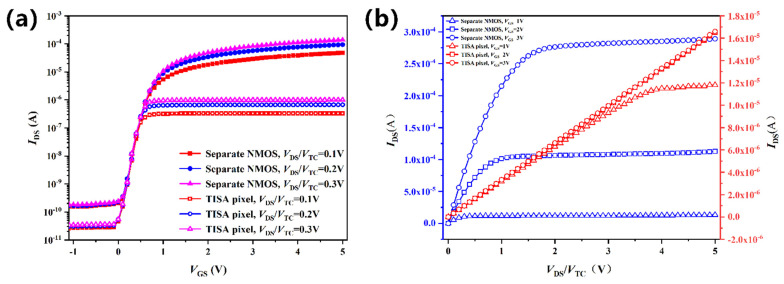
(**a**) *I*_D_-*V*_DS_/*V*_TC_ and (**b**) *I*_D_-*V*_GS_ characteristic curves of a TISA pixel and a separate NMOS.

Figure 6 compared the *I*_DS_-*V*_DS_/*V*_TC_ and *I*_D_-*V*_GS_ characteristics curves of the TISA pixel and a separate NMOS device. In practice, in the condition that *V*_DS_ was equal to *V*_TC_, due to a voltage drop of the thermopile, the drain voltage of NMOS in the TISA pixel was much lower than *V*_TC_, which resulted in a fall in *I*_DS_. Meanwhile, *I*_DS_-*V*_GS_ characteristic curves of the TISA pixel had a relative downward shift and the triode region of the *I*_DS_-*V*_TC_ curve had an extension to the right along the *x*-axis.

**Figure 7:**
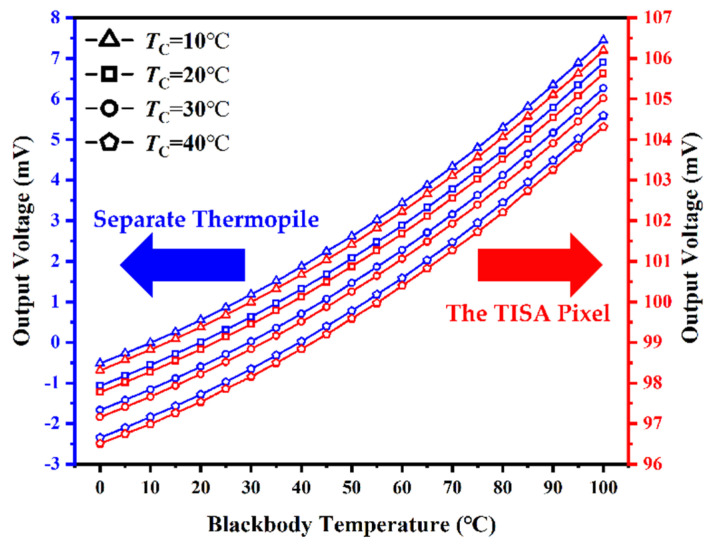
Output voltage curves of a separate thermopile and a TISA pixel at different chamber temperatures (*T*_C_) with increasing blackbody temperature.

In order to evaluate the effect of ambient temperature on the devices’ performance, the sensitivity of the TISA pixel and a separate thermopile were measured with the system shown in Figure 4. In the test system, *V*_GS_ and *V*_TC_ were supplied at 5 V and 100 mV, respectively, to ensure that the NMOS in the TISA pixel operated in the triode region. Moreover, the constant temperature chamber (*T*_C_) was set to 10 °C, 20 °C, 30 °C and 40 °C and the blackbody provided a temperature range of from 0 °C to 100 °C. the output voltage–temperature of the blackbody curves of t TISA pixel and the separate thermopile with increasing temperature of the blackbody are shown in Figure 7. From the acquired data, it can be observed that the output voltages of the TISA pixel and the separate thermopile are relatively consistent. Additionally, with the decline of *T*_C_, the output voltage curves both move forward along the *Y*-axis. This is because the thermopile is a temperature-difference infrared detector. In the thermopile, the temperature of the cold junction is related to the ambient temperature, and the temperature of the hot junction is related to the target temperature. As ambient temperature drops, at the same temperature of the blackbody, the temperature difference between the cold junction and the hot junction increases, resulting in a larger output voltage of the thermopile and vice versa. Thus, due to the similar output voltage characteristics, subsequent readout circuits of the separate thermopile and the TISA pixel could both be calibrated by negative temperature coefficient (NTC) thermistor for ambient temperature compensation. Meanwhile, according to the obtained relationship between the output voltage and the blackbody temperature, the responsivity ($R_{v}$) of the TISA pixel can be calculated as 94.5 V/W and the noise equivalent temperature difference (NETD) can be calculated as 109 mK.

**Figure 8:**
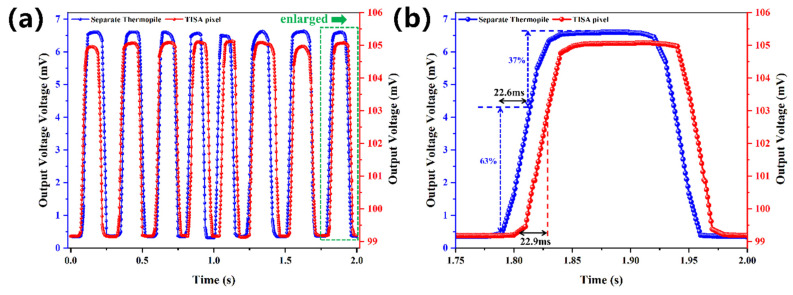
(**a**) Response voltages of the TISA pixel and the separate thermopile under a blackbody radiation of 100 °C and a chopper frequency of 4 Hz in 2 s, (**b**) One enlarged period of the response waveform.

Figure 8a shows the response voltages of the TISA pixel and the separate thermopile under a blackbody radiation of 100 °C and a chopper frequency of 4 Hz in 2 s. When the TISA pixel was measured, 5 V and 100 mV voltages were applied at *V*_GS_ and *V*_TC_, respectively, which ensured that the NMOS switch was at the status of ON. Figure 7b shows the one enlarged period of the response waveform in Figure 7a. Response times for the separate thermopile and the TISA pixel were 22.6 ms and 22.9 ms. The integrated NMOS switch does not extend the response time of the thermopile.

**Figure 9:**
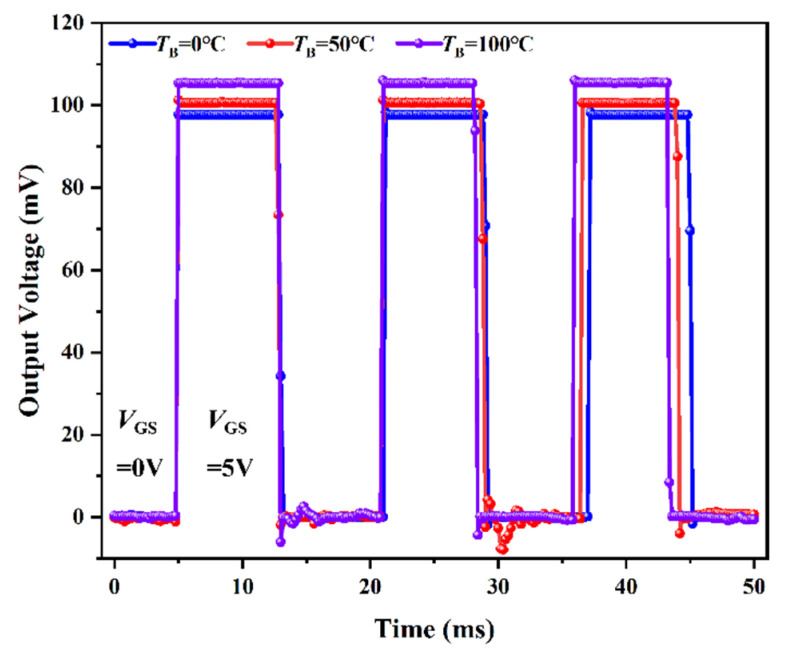
Readout of *V*_OUT_ in the TISA pixel controlled by *V*_GS_ of NMOS at different blackbody temperatures (*T*_B_).

The readout of *V*_OUT_ in the TISA pixel can be rapidly controlled by V_GS_ at different temperatures of the blackbody, as shown in Figure 9. The measuring frequency of the multimeter was set at 5 kHz. It can be observed that some data cannot be collected on the rising or falling edge of *V*_OUT_ jump. Therefore, it is assumed that the response time of the NMOS as a switch is less than 200 μs. Thus, in a TISA, the NMOS can be used as a switch to ensure that *V*_OUT_ of each pixel is addressed in order, rapidly and effectively.

**Figure 10:**
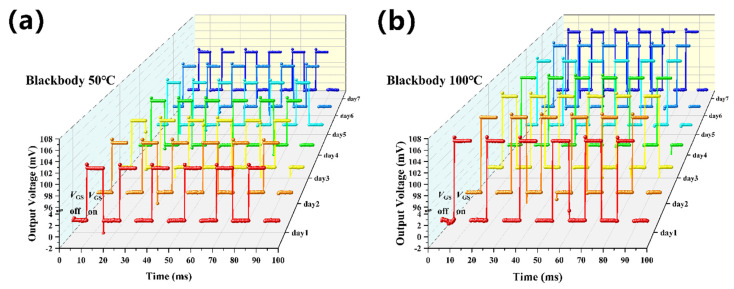
A one−week reliability test at blackbody temperatures of 50 °C (**a**) and 100 °C (**b**) respectively.

Figure 10 shows the stability test results of the TISA pixel. Over the course of one week, each day the sensor was measured at blackbody temperatures of 50 °C and 100 °C, respectively. The experimental results showed that the sensing and the switch performance of the TISA pixel remained consistent over time. Benefiting from the stability of the standard CMOS processes and the applied materials, the TISA pixel has good repeatability and reliability.

## 5. Conclusions

In this paper, a TISA pixel, monolithically integrated with an NMOS, is presented. By interleaved MEMS and CMOS processing in a single process sequence, the pixel is fabricated. The CMOS compatible monolithic integration method can effectively reduce the total number of masks and the product cycle. Meanwhile, compared with a separate thermopile, the thermopile integrated with an NMOS in the TISA pixel retains a fast response, large sensitivity and high reliability. In addition, the gate voltage of NMOS can rapidly and effectively control the readout of thermopile output voltage, which facilitates the addressing of each pixel in the array. These results demonstrate that the TISA pixel fabricated by the monolithic integration process is feasible, low-cost, and high-performance and is expected to have a wide range of commercial demands.

## Figures and Tables

**Table 1:** Role of each process and each mask.

	Processes	Lithography? (Y/N)	Key Parameters	For Thermopile	For NMOS
1	SiO_2_ by wet oxidation	Y	500 nm	Supporting layer	Ion implantation barrier
2	Si_3_N_4_ deposition	Y	100 nm	Supporting layer	-
3	SiO_2_ by dry oxidation	Y	30 nm	-	Dielectric layer of gate
4	Poly-Si deposition	Y	300 nm	N-poly-Si thermocouple strips	Gate
5	Phosphorus ions implantation	N	Dose: 1 × 10^16^ cm^−2^ Energy: 50 KeV	Forming n-poly-Si thermocouple strips	Forming source, drain and gate
6	SiO_2_ deposition	Y	200 nm	Insulator	
7	Poly-Si deposition	Y	300 nm	P-poly-Si thermocouple strips	-
8	Boron ions implantation	N	Dose: 2 × 10^16^ cm^−2^ Energy: 50 KeV	Forming p-poly-Si thermocouple	Forming bulk contact
9	RTA	N	950 °C, 30 s	Activating injected ions	
10	SiO_2_ deposition	Y	200 nm	Insulator	
11	Al sputtering	Y	500 nm	Metal interconnection	
12	SiO_2_ deposition	Y	200 nm	Thermal conductance for hot junctions; Thermal insulation for cold junctions	-
13	Si_3_N_4_ deposition	Y	500 nm	Absorption layer	Passivation layer
